# Mass Spectrometry Imaging for Glycome in the Brain

**DOI:** 10.3389/fnana.2021.711955

**Published:** 2021-07-29

**Authors:** Md. Mahmudul Hasan, Mst. Afsana Mimi, Md. Al Mamun, Ariful Islam, A. S. M. Waliullah, Md. Mahamodun Nabi, Zinat Tamannaa, Tomoaki Kahyo, Mitsutoshi Setou

**Affiliations:** ^1^Department of Cellular & Molecular Anatomy, Hamamatsu University School of Medicine, Hamamatsu, Japan; ^2^International Mass Imaging Center, Hamamatsu University School of Medicine, Hamamatsu, Japan; ^3^Department of Systems Molecular Anatomy, Institute for Medical Photonics Research, Preeminent Medical Photonics Education & Research Center, Hamamatsu, Japan

**Keywords:** glycans, glycosylation, mass spectrometry imaging, brain tissue, three-dimensional MSI

## Abstract

Glycans are diverse structured biomolecules that play crucial roles in various biological processes. Glycosylation, an enzymatic system through which various glycans are bound to proteins and lipids, is the most common and functionally crucial post-translational modification process. It is known to be associated with brain development, signal transduction, molecular trafficking, neurodegenerative disorders, psychopathologies, and brain cancers. Glycans in glycoproteins and glycolipids expressed in brain cells are involved in neuronal development, biological processes, and central nervous system maintenance. The composition and expression of glycans are known to change during those physiological processes. Therefore, imaging of glycans and the glycoconjugates in the brain regions has become a “hot” topic nowadays. Imaging techniques using lectins, antibodies, and chemical reporters are traditionally used for glycan detection. However, those techniques offer limited glycome detection. Mass spectrometry imaging (MSI) is an evolving field that combines mass spectrometry with histology allowing spatial and label-free visualization of molecules in the brain. In the last decades, several studies have employed MSI for glycome imaging in brain tissues. The current state of MSI uses on-tissue enzymatic digestion or chemical reaction to facilitate successful glycome imaging. Here, we reviewed the available literature that applied MSI techniques for glycome visualization and characterization in the brain. We also described the general methodologies for glycome MSI and discussed its potential use in the three-dimensional MSI in the brain.

## Introduction

Glycans are diverse and complex structured sugar chains and crucial to many biological processes in all living organisms. Organisms polymerize simple sugars to process oligo- and polysaccharides, commonly referred to as glycans, when freely or covalently linked to proteins and lipids. Glycans are made up of several sugar residues with distinct glycosidic bonds, forming a complex, branched structure. As a result, various glycan structures have been generated in organisms with different glycosylation forms (Cipolla et al., 2011; Coff et al., 2020). Glycans are covalently linked to proteins and modulate their functions through direct interactions that control protein conformation, stabilization, and turnover. During protein biosynthesis, amino acids may be modified by the covalent binding of several functional groups. This protein modification type is known as post-translational modification (PTM) (Veillon et al., 2018). The glycosylation of proteins and lipids occurs in the endoplasmic reticulum (ER) and the Golgi apparatus of the cells.

In contrast to the genome and proteome, glycans are generated in non-templated processes and are carefully regulated at different levels in ERs and Golgi apparatuses (Reily et al., 2019). There are also no “completed” glycan structures. During the glycosylation, proteins may be modified, resulting in various glycans that differ uniformly by interactions, length, number of antennas, and composition (Ruhaak et al., 2018).

In glycosylation, carbohydrates are linked to a protein (as N-linked or O-linked glycans), lipid, or glycan substrate (Iqbal et al., 2018; Mueller et al., 2018). In the protein glycosylation processes, asparagine, serine, threonine, tryptophan (Ihara, 2015.), hydroxyproline, hydroxylysine (Song and Mechref, 2013), and rarely tyrosine residues are connected by the binding with carbohydrate moieties, resulting in a mass increase of the substrate protein. The glycosylation of proteins and lipids is known to be associated with brain development, cell adhesion, molecular trafficking, signal transduction, and differentiation (Ohtsubo and Marth, 2006). Aberrant glycosylation including, chemical modification, cutting or elongation of glycan structures on the cellular surface, critical receptors on the cell surface, and neurotransmitter transporters, are associated with various neurological abnormalities, including immune responses, neurodegenerative disorders, mental disorders, and brain tumors (Ohtsubo and Marth, 2006; Hwang et al., 2010; Iqbal et al., 2018). It is indispensable to understand the intrinsic molecular mechanisms, cellular profiling, and glycome imaging to determine the brain function and behavior in neurological disorders associated with glycosylation. Chemical reporters and glycan-binding proteins such as lectins and antibodies techniques have been typically used for specific glycan determination and imaging. However, those traditional approaches for glycan imaging are inadequate to cover the wide range of mammalian glycans (Eshghi et al., 2014). Therefore, it is essential to use alternate imaging methods to complement the detailed information acquired from histostaining methods.

Mass spectrometry imaging (MSI) has emerged as a method of choice for reliable brain imaging to study the roles of glycosylation in the brain. MSI has overcome some of the difficulties of conventional histostaining methods due to its high ionization efficiency. Unlike traditional affinity-based detection methods such as immunohistochemistry, MSI techniques do not require prior information from the analytes of interest. This feature, which is a unique characteristic of MSI, is especially useful for discovery study (Eshghi et al., 2014). MSI is a unique method that incorporates histology and mass spectrometry (MS). It allows label-free detection of hundreds to thousands of compounds in a single tissue section without extraction, purification, or separation (Eriksson et al., 2013; Buck et al., 2015). In addition, MSI can combine quantitative techniques that may allow for direct quantitative imaging of various analytes from a tissue section. Recent developments in MS instrumentations, methods, and data analysis have propelled quantitative glycomics studies to greater levels (Zaia, 2008). Matrix-assisted laser desorption ionization-mass spectrometry imaging (MALDI-MSI), desorption electrospray ionization mass spectrometry imaging (DESI-MSI), and secondary ion mass spectrometry (SIMS) imaging are three of the most popular ionization technologies used in MSI (McDonnell and Heeren, 2007). So far, MALDI-MSI has been applied to visualize glycome due to its higher sensitivity, ionization efficiency and high mass accuracy over a broad mass-to-charge (*m/z*) range.

The capability of MSI to capture and visualize spatial distributions of biomolecules such as proteins (Heijs et al., 2015; Piehowski et al., 2020), lipids (Wildburger, 2017; Islam et al., 2020), glycans (Mori et al., 2019; Heijs et al., 2020), and metabolites (Cornett et al., 2008; Sugiura and Setou, 2010) in brain tissues with high mass accuracy and resolution enables the identification of compounds through accurate mass matching. Therefore, it is essential to further MSI application knowledge and accelerate medical diagnostics development (Neubert and Walch, 2013; Schubert et al., 2016). MSI platforms have witnessed rapid growth, and they now can provide comprehensive chemical information at subcellular spatial resolution (Zavalin et al., 2012). Current technological developments in instrumentation and software have made MSI an analytical tool capable of identifying and characterizing a wide range of molecular species while simultaneously imaging their spatial distributions with accurate mass measurements. This ability of simultaneous molecular detection, spatial imaging, and relative abundance of biomolecules helps MSI applications in the research field of brain glycobiology. Currently, it has been shown that glycans released from glycoconjugates that have been immobilized in a solid phase could be examined directly by MSI techniques.

Here we have focused mainly on the methodologies of glycome imaging in animal and human brains using MSI techniques. In addition, we have discussed the limitations of the current methodology and the future development for the study of glycome by MSI.

## Role of Glycosylation in the Brain

Glycosylation is a common enzymatic mechanism in which a carbohydrate donor is glycosidically linked to a functional group of another molecule (proteins or lipids) that produces glycoconjugates. In the protein and lipid glycosylation process, oligosaccharides are attached to proteins or lipids such as N-linked or O-linked glycans. In the consensus sequence of Asn-X-Ser/Thr, N-glycans are linked to the nitrogen of the amide-side chain in the asparagine residue, where X can be any amino acid other than proline. On the other hand, O-glycans are covalently attached to the oxygen of the hydroxyl group of serine, threonine, or tyrosine (Van Den Steen et al., 1998; Imperiali and O’Connor, 1999). Glycosylation is a ubiquitous PTM of proteins in all three domains of life, which plays a vital role in determining protein structure, function, and stability (Moremen et al., 2012). Glycolipids are also synthesized through this process in eukaryotes and prokaryotes and play diverse biological roles (Kopitz, 2017). Approximately 700 proteins are associated with glycosylation in mammals. Among them, about 200 proteins are glycosyltransferases (Moremen et al., 2012). These proteins play a critical role in producing glycoconjugates possessing diverse functions in several organs in mammals, including the brain. About 70% of brain proteins are present as glycoproteins. They mainly contain a higher number of high-mannose, hexose, and N-acetyl hexosamine (HexNAc) with fucose and sialic acid (Lee et al., 2020; Tena and Lebrilla, 2021). Many brain lipids also present as glycoconjugates that play various biological functions in the brain (Tena and Lebrilla, 2021).

Glycosylation has pivotal roles in brain development, physiology, and functions, including regulating synaptic processes and neural excitability by controlling neural transmission (Scott and Panin, 2014; Klarić et al., 2020). In the brain, neurons, oligodendrocytes, and astrocytes are developed from neural stem cells, which depend on cell surface molecules present on the neural stem cells and their interactions with other molecules and cells (Yale et al., 2018). Moreover, cells derived from neural stem cells also depend on their interaction with other cells and molecules through glycosylation (Yale et al., 2018). Therefore, dysregulated glycosylation can develop several neurological disorders. For example, N-glycosylation is involved in developing the human nervous system, which controls the folding, trafficking, localization, adhesion, cell-cell interaction, and enzymatic activity of proteins (Freeze et al., 2012). O-glycosylation also regulates the functions of thousands of proteins in the brain and significantly affects neuroprotection, memory function, and neuronal signaling. Impaired biosynthesis of glycoconjugates due to altered N- or O-glycosylation can cause several neurological diseases, including cerebellar atrophy, stroke, epilepsy, neuropathy, delayed development, paralysis, and tremors (Freeze et al., 2012; Reily et al., 2019). The analysis of post-mortem brain samples shows that people with schizophrenia have aberrant glycosylation (Williams et al., 2020). Dysregulated fucosylation and sialylation of both N- and O-glycans also play a critical role in brain cancer development and progression (Veillon et al., 2018). Aberrant glycosylation of proteins associated with inhibitory and excitatory neurotransmission, including glutamate transporter, gamma-aminobutyric acid receptors, and quisqualate receptors, was recently reported in individuals with schizophrenia.

Additionally, differential expression of glycosyltransferases was observed in a study using the post-mortem brain of schizophrenia patients (Williams et al., 2020). Zhang et al. have reported the N-glycoproteome profile of Alzheimer’s disease (AD) and the unaffected brains using mass spectrometry-based proteomics analysis. This study revealed dysregulated N-glycosylation mediated altered pathways in human AD brains, including synaptic dysfunction, altered lysosomal function, dysregulated cell adhesion, and cell signaling (Zhang et al., 2020). As well as proteins, lipids glycosylation also has pivotal roles in the development of the central nervous system (CNS), synaptic plasticity, and regeneration, and its dysregulation causes several neurological disorders. Glycolipids are the cell membrane’s structural components and act as ligands for signaling molecules, control cell-to-cell communication, and form lipid rafts. The most common glycolipids are glycoglycerolipids, cerebrosides, and gangliosides (Brandenburg and Holst, 2015). They are abundant in eukaryotic cells, including, neuronal and glial cells of the CNS, and play a crucial role in controlling these cell’s functions (Brekk et al., 2020). Altered glycolipids in the brain due to dysregulated glycosylation can cause several neurodegenerative diseases, including Parkinson’s disease, AD, Huntington’s disease, and amyotrophic lateral sclerosis in humans (Brekk et al., 2020; Moll et al., 2020).

Although glycobiology has opened a new window for glycan-based therapeutics for several diseases such as breast cancer and human immunodeficiency virus (HIV-1), glycan-based therapy for neurological diseases is yet to be developed (Doran et al., 2018; Reily et al., 2019). Substantial benefits must be achieved for neurological disorders of glycosylation by early detection and management. Hopefully, further development in glycobiology can offer better therapeutic approaches for treating neurological disorders in the future.

## Imaging Techniques for the Brain Glycome

Glycan-binding proteins, including lectins and antibodies, have traditionally been used to recognize and imaging glycans or glycoconjugate complexes in brain tissues. Lectins are naturally occurring proteins identified in most organisms (Lis and Sharon, 1998) and have been widely used for the detection (Pilobello and Mahal, 2007) and enrichment (Hirabayashi, 2004) of glycoconjugates. These naturally occurring lectins are also used to research brain disorders based on glycan expression (Gabius, 2009). The significant biological glycan residues such as sialic acids, mannose, fucose, and galactose can be identified from wheat germ agglutinin (WGA), Concanavalin A; Lens culinaris agglutinin, Aleuria aurantia lectin, and peanut agglutinin (PNA); Ricinus communis Agglutinin; Griffonia simplificola lectin-I lectins, respectively. WGA and cholera toxin B (CTB) is a neuronal tracer based on lectin that is endocytosed readily by brain cells after binding to glycolipids of the surface in the cells (Jobling et al., 2012), making them helpful in understanding the complex and functional neuronal network connections in both anterograde and retrograde directions (Parker et al., 2013, 2015; Lee et al., 2017). WGA tracer has been used in the mouse brain tissue to study neuronal synapses (Yoshihara, 2002) and for transneuronal tracing purposes in several animal species, including Drosophila (Tabuchi et al., 2000) and rodent brains (Libbrecht et al., 2017). Glycans were visualized using lectins on tissue sections during mouse secondary neurulation (Griffith and Wiley, 1989) and human thymus (Paessens et al., 2007). Although lectins have contributed significantly to glycosylation profiles and neural networks in the brain, they appear to have a low affinity for their glycan epitope and require multi-valence for high-avidity binding (Kiessling and Pohl, 1996). In addition, lectins are usually tissue-impermeable, and sometimes they are toxic (Schwarz et al., 1999; Ohba and Bakalova, 2003).

Previously, antibodies against protein antigens have been developed and characterized against several glycan structures for particular neuronal cells (Jacobsen et al., 2016). These functional monoclonal anti-glycan antibodies remain elusive due to the difficulties experienced throughout their choice and development (Sterner et al., 2016). The most commercially available anti-glycan antibodies are IgM and IgG subtypes, used for imaging of O-glycans, N-glycans, and so on (Cummings and Etzler, 2009). Generally, they have high glycan epitopes specificity and affinity. Like lectins, antibodies have limited permeability in tissues and have been used for specific glycans (Laughlin and Bertozzi, 2009).

Biorthogonal chemical reporter techniques have been used to target and imaging sialoglycans in living organisms (Laughlin et al., 2008; Cheng et al., 2016). This method uses endogenous metabolic tracers that consist of sialic acid analogs or N-acetylmannosamine (ManNAc) linked with chemical reporters such as azide. *In vivo* brain imaging of these glycans manipulation techniques has been reported recently (Gagiannis et al., 2007; Sampathkumar et al., 2008; Xie et al., 2016; Shajahan et al., 2017). However, it is difficult to monitor the sialoglycans in the brain tissues because of the labeled glycan’s impermeability through the blood–brain barrier (BBB). In order to address this problem, carrier-mediated transport systems have been developed. For example, modified ManNAc conjugated with neuroactive carriers including choline present in BBB mediated access into mice brain by intravenous injection (Shajahan et al., 2017). Although the techniques above are advantageous for brain glycan imaging, there are some drawbacks: they are only used for a limited number of glycan targets, they are not labeled free, and spontaneous imaging methods are often toxic.

MSI is an emerging tool that offers label-free imaging of the tissue glycome allowing the detection of several glycans at a time, with the spatial distribution and regional heterogeneity with accurate mass matching. Thus, some of the difficulties of histological staining with lectins, antibodies, and chemical reporters can be resolved (Iqbal et al., 2018).

## Overview of MSI

The MSI technique consists of an ionization source, the mass analyzer, and the detector. A specimen is examined/scanned under ambient conditions or vacuum using a light beam, lasers, or ions; as a result, analytes are ionized and desorbed simultaneously. The most commonly used ionization methods for MSI are MALDI, DESI, secondary ion beam, and laser ablation post-ionization. The selection of an appropriate ionization approach is crucial to ionize the specific analyte of interest efficiently. After the ionization process, the next step is to transfer them into the mass analyzer for separation and characterization. Various mass analyzers have been coupled to MSI, including time-of-flight (TOF), TOF/TOF, quadrupole TOF mass analyzers (QTOF), linear ion trap, Orbitrap, and Fourier-transform ion cyclotron resonance (FT-ICR), and they offer a varying degree of mass resolution, accuracy, and speed. Typically, MALDI is integrated into TOF systems, offering a mass error of <10 ppm and mass resolution up to 50,000. The TOF/TOF system offers MS/MS facilities with a mass resolving power of >60,000 and a mass error of <1 ppm. The TOF/TOF system currently offers rapid and highly reproducible fragmentation with structural characterizations of glycome formulated to simplify the linkage-specific substituent information on the terminal units (Mechref et al., 2003; Yu et al., 2006). These benefits are often missing in traditional TOF or QTOF systems. The QTOF mass analyzer provides the MS/MS capability (Loboda et al., 2000), while the Orbitrap combined with MALDI-MSI provides up to 100,000–200,000 mass resolution, <5 ppm mass error, and MS/MS capability (Makarov, 2000; Zubarev and Makarov, 2013). FT-ICR combined with MALDI offers the ultrahigh mass-resolving power (m/*upDelta*m_50%_ > 27,00,000 at *m/z* 400) and mass accuracy (80 ppb RMS) that enable confident identification of tens of thousands of unique elemental compositions (Marshall, 2000; Smith et al., 2018).

Spatial resolution in MALDI-MSI depends on four major factors: (1) analyte delocalization during sample preparation, (2) matrix homogeneity, (3) laser spot size, and (4) sensitivity (Dueñas et al., 2016). Sensitivity has become a critical factor that has improved by advanced sample preparation and instrumentation. Advanced technologies have been utilized to improve spatial resolution, usually ranging from 50 to 200 μm for tissue MSI analysis (Römpp and Spengler, 2013). The high-quality protein images have been achieved at 5 μm spatial resolution using a Gaussian laser beam and an aspheric lens in MALDI-FT-ICR MSI (Zavalin et al., 2014). Using transmission geometry MALDI-TOF-MSI technology, up to 1 μm spatial resolution images have been achieved in mouse brain tissue analyses (Zavalin et al., 2012). Similarly, by modifying the beam-delivery optics, up to 5 μm spatial resolution has been achieved by the Orbitrap in biological tissues (Korte et al., 2015).

Of the most frequently used MSI techniques, TOF and Orbitrap systems are attractive for high throughput analysis, whereas TOF/TOF and FT-ICR systems are more suitable for discovery and research platforms. After the ions are separated based on their *m/z* ratio in the analyzer, ions need to be detected. The detector converts charged ions/current flow into electric signals digitally. The commonly used detectors are the Faraday cup, photographic plate, an electron multiplier (Potts, 1987). Nowadays, an image current detector is used in modern mass spectrometers with FT-ICR and orbitrap (Wu et al., 2020).

Molecular detection also depends on the sensitivity of the MSI instruments. While DESI and SIMS offer better sensitivity for the low mass molecules (<500 Da), MALDI has greater sensitivity for the high mass molecules (>500 Da) (Nabi et al., 2021). The recent addition of MALDI-2 MSI and 21 tesla MALDI-FT-ICR techniques offers the highest sensitivity, mass resolution, accuracy, spatial resolution, molecular details, dynamic range, and MS/MS capabilities (Smith et al., 2018; Niehaus et al., 2019; Bowman et al., 2020; Heijs et al., 2020). On the other hand, on-tissue treatment with buffer-free phospholipase C reduced the ion suppression effects, thereby ion signal intensities of mono-, di-, and oligohexosylceramides were enhanced by up to 10-fold in flash-frozen (FF) tissue sections without decreasing the high lateral resolution of MSI analyses (Vens-Cappell et al., 2016). These latest MSI instrumentations and methodologies may improve the glycome detection, characterization, and visualization in biological tissues.

## Methodology for Glycome MSI in the Brain

Careful sample preparation is crucial for successful MSI analysis of glycomics from a thin tissue section. Therefore, a series of laborious and delicate sample pre-treatment is required as protein denaturation, deglycosylation, stabilization for efficient glycome MSI in the tissue section.

**FIGURE 1:**
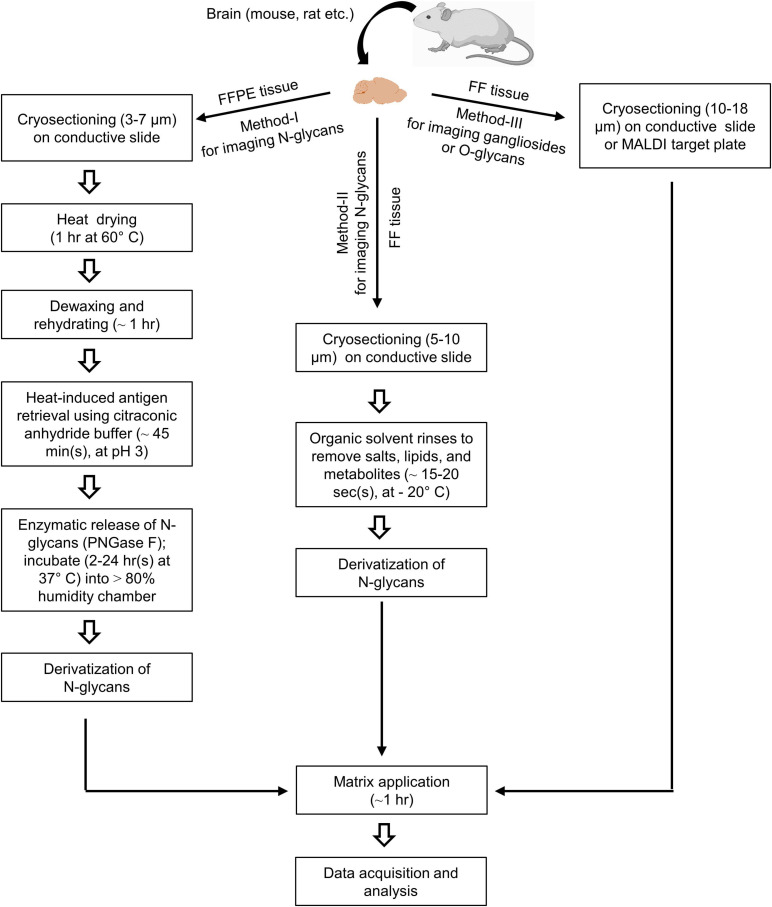
Schematic workflow of mass spectrometry imaging (MSI) for glycome in the brain. An FFPE tissue section is mounted on an indium tin oxide-coated (ITO-coated) glass slide and dried by heating. Then section is deparaffinized and rehydrated by xylene and a series of ethanol rinsing, respectively. The epitope activation and tissue proteins are denatured by the treatment with a basic heat-induced antigen retrieval buffer (citraconic anhydride buffer, at pH 3) (Method-I). On the other hand, FF tissue (Method-II) rinses in an organic solvent for removing salts, lipids, and metabolites. Another FF tissue is directly mounted on the conductive ITO-coated glass slide or MALDI target plate for matrix spraying and MSI analysis (Method-III). Enzymatic release of N-glycans is performed using PNGase F under incubation at 37°C (Method-I). On-tissue derivatization is required (Method-I and II) before matrix deposition to stabilize glycans and enhance the ionization efficiency. After automated matrix spraying, the sections are subjected to a laser source for data acquisition and analysis.

Formalin-fixed paraffin-embedded (FFPE) tissue should be cryosectioned (∼3–7 μm) and mounted on a conductive glass slide such as indium tin oxides-coated slides (ITO-coated) (Figure 1, method-I). On the other hand, fresh-frozen or FF tissue should be cryosectioned (∼5–10 or ∼5–18 μm) and mounted on a conductive slide or MALDI target plate to achieve high-resolution ion images (Figure 1, method-II and III). Then mounted FFPE tissue proteins should be denatured by dry heating (1 h at 60°C) (Drake et al., 2018). FFPE tissue must be dewaxed properly to remove paraffin, which may obscure the N-glycan signal. Deparaffinization of FFPE tissue includes washing in xylene solution and rehydrating it in a series of ethanol rinses at room temperature. In addition, FF tissue must be rinsed in organic solvents (e.g., CHCl_3_) to remove salts, lipids, and other metabolites to improve N-glycans detection significantly (Yang and Caprioli, 2011). MSI of gangliosides can be achieved from FF tissues without enzyme pre-treatments and derivatization (Whitehead et al., 2011; Jackson et al., 2018). After dewaxing FFPE tissue, heat-induced antigen retrieval should be performed using citraconic anhydride buffer into a pre-warmed device to break protein crosslinks (Drake et al., 2018). Antigen retrieval breaks chemical crosslinking formed by formalin, allowing for efficient digestion. It is crucial for unmasking hidden or latent epitopes in preparation and allows for enzymatic access for releasing glycans from the proteins in the tissue. For FF tissue sections, antigen retrieval is not necessary (Raghunathan et al., 2019).

**FIGURE 2:**
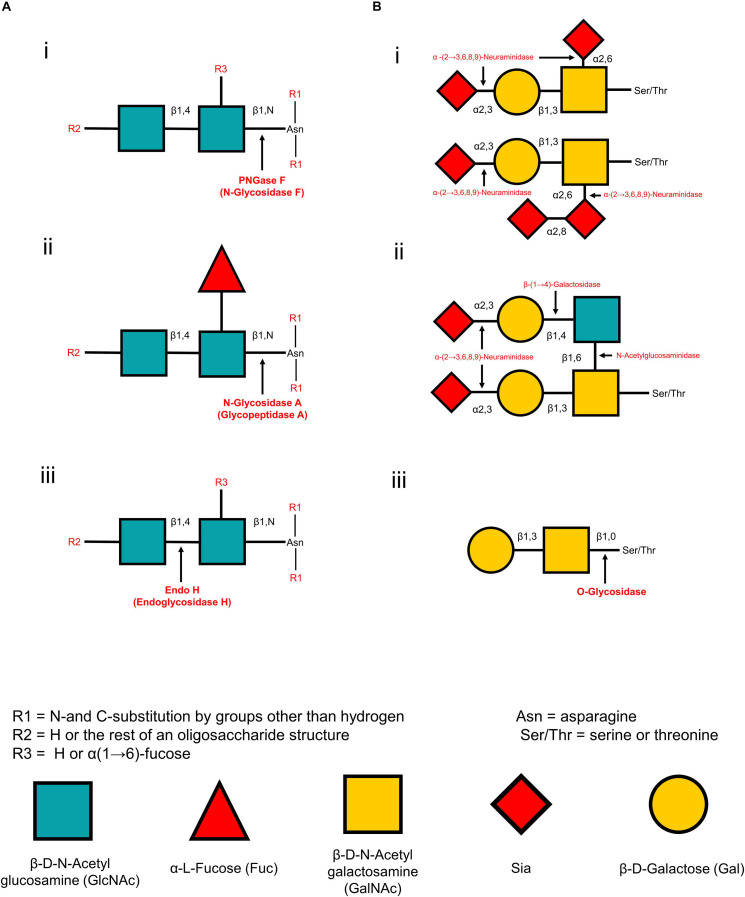
Cleaving sites for enzymatic deglycosylation of glycans. N-glycan strategies **(A)** and O-glycan strategies **(B)**. PNGase F cleaves all asparagine-linked complex, hybrid, or high mannose oligosaccharides unless the core contains an α(1→3)-fucose. Cleaving site for PNGase F **(A-i)**. N-glycosidase A hydrolyzes oligosaccharides containing a fucose residue α(1→3)-linked to the asparagine-linked N-acetylglucosamine **(A-ii)**. Endo H cleaves the β-1,4 glycosidic linkage of high-mannose N-linked glycans between GlcNAc1 and GlcNAc2 **(A-iii)**. In a sequential glycolytic cleavage, disialylated and trisialylated O-linked glycans have the sialic acid residues (NeuNAc) removed by α(2→3,6,8,9)-neuraminidase to release core-1 type O-glycans **(B-i)**. Disialylated O-linked Core-2 hexasaccharide or sialic acid residues are sequentially degraded by α(2→3,6,8,9)-neuraminidase, β(1→4)-galactosidase and N-acetylglucosaminidase to release core-1 type O-glycans **(B-ii)**. Cleavage site for O-glycosidase on the core-1 type O-glycans after sequential glycolytic degradation **(B-iii)**.

The release of glycans from glycoproteins is crucial and facilitates its analysis by increasing sensitivity and simplicity. The methods for releasing glycans vary between N-linked and O-linked glycans and can be divided into chemical and enzymatic reactions (Table 1). The chemical methods can release both N-linked and O-linked glycans, but those methods may degrade the released glycans and proteins, forming other by-products (Koutsioulis et al., 2008). Hence, the enzymatic release of glycans is widely applied for MSI analysis. Peptide-N-glycosidase F (PNGase F) is a conventionally used enzyme to release N-glycans (Figure 2A-i). It is an amidase enzyme that dissociates N-glycans and asparagine residues from the innermost N-acetylglucosamine (GlcNAc). The PNGase F is used to release high mannose, complex, and hybrid N-glycans due to its broad specificity. The only limitation for such enzymes is that one cannot cleave if an α(1→3)-fucose is linked to the core GlcNAc residue (Veillon et al., 2017). Glycopeptidase A hydrolyzes oligosaccharides containing a fucose residue α(1→3)-linked to the core GlcNAc residue (Figure 2A-ii). Endoglycosidase H cleaves the β-1,4 glycosidic linkage of high-mannose N-linked glycans between GlcNAc1 and GlcNAc2 residues (Figure 2A-iii). They are also known as chitobiose. While most N-linked oligosaccharides can be removed using PNGase F, there are no universal and efficient enzymes for releasing O-linked glycans. For example, endo-α-N-acetylgalactosaminidase only cleaves O-glycans with a core-1 such as β(1→3)-linkage to the GalNAc structure. Another novel O-glycosidase has a slightly broader specificity, cleaves both the core-1 and then core-3 structures (Koutsioulis et al., 2008; Guthrie and Magnelli, 2016). In another way, monosaccharides must be sequentially hydrolyzed by a series of exoglycosidases until only the Gal-β(1→3)-GalNAc core remains. α(2→3,6,8,9)-neuraminidase enzyme is capable of efficient cleavage of the NeuAc-α(2→8)-NeuAc bond (Figure 2B-i). Disialylated (NeuNAc) O-linked core-2 hexasaccharide is sequentially degraded by using α(2→3,6,8,9) neuraminidase (Figure 2B-i). β(1→4)-galactose (Gal) residues are removed by β(1→4)-galactosidase, and N-acetylglucosamine (GlcNAc) residues are cleaved by N-acetylglucosaminidase (Figure 2B-ii). Finally, O-glycosidase (endoglycosidase) hydrolyzes the serine or threonine-linked unsubstituted O-glycan core [Gal-β(1→3)-GalNAc] (Figure 2B-iii). Any type of modification in the core structure can stop the action of O-glycosidase. These enzymes can be applied on the tissue of interest by an automated sprayer.

**TABLE 1:** Chemical and enzymatic deglycosylation methods for glycans.

N-glycans	Conditions	References
**Chemical release**		

Anhydrous hydrazine	Incubation at 100°C for 8–12 h(s)	Takasaki et al., 1982; Patel et al., 1993
Oxidation using sodium hypochlorite	Shaking incubation at 37°C for 1 h	Song et al., 2016

**Enzymatic release**		

PNGase F from *Flavobacterium meningosepticum*	Incubation at 37°C for 2 h(s) to overnight	Tarentino et al., 1985; Drake et al., 2018
PNGase A purified from almond *Prunus amygdalus* var. *dulcis*	Incubation at 37°C for 1–24 h(s)	Tretter et al., 1991
Endoglycosidase F1, F2, F3 (purified from *Elizabethkingia miricola*)	Incubation at 37°C for 1–18 h(s)	Trimble and Tarentino, 1991; Tarentino and Plummer, 1994; O’Neill, 1996
Endoglycosidase H (Endo H) purified from *Streptomyces plicatus*	Incubation at 37°C for 1–24 h(s)	Tarentino et al., 1974; Maley et al., 1989; Freeze and Kranz, 2010
Endo-β-N-acetylglucosaminidase (Endo-M) purified from *Mucor hiemalis*	Incubation at 30°C for 20 h(s)	Fujita et al., 2004
Endo-β-N-acetylglucosaminidase D (Endo-D) from *Streptococcus pneumoniae*	Incubation at 37°C for 1 h	Yamamoto et al., 2005
Endo-β-N-acetylglucosaminidase FV (Endo FV) purified from *Flammulina velutipes*	Incubation at 37°C for 14 h	Hamaguchi et al., 2009
Endoglycosidase S from *Streptococcus pyogenes*	Incubation at 37°C for 30 min(s)	Collin and Olsén, 2001; Trastoy et al., 2018

**O-glycans**		

**Chemical release**		

Anhydrous hydrazine	Incubation at 75°C for 16 h(s)	Patel et al., 1993
Alkaline-β-elimination	Incubation at 55°C for 18 h(s)	Walker et al., 2003
Non-reductive β-elimination	Incubation at 60°C for 20 h(s)	Furuki et al., 2017
Oxidation using sodium hypochlorite	Incubation at room temperature for 24 h(s)	Song et al., 2016

**Enzymatic release**		

α-(2→3,6,8,9)-Neuraminidase from *Arthrobacter ureafaciens*	Incubation at 37°C for 5 min(s) to 1 h	Cabezas, 1991
β-(1→4)-Galactosidase from *Streptococcus pneumoniae*	Incubate at 37°C for 1 h	Buckeridge and Grant Reid, 1994
N-Acetylglucosaminidase from *Talaromyces emersonii*	Incubation at 37°C for 4 h(s)	Chai et al., 1997; Saldova and Wilkinson, 2020
Endo-α-N-acetylgalactosaminidase from *Clostridium perfringens*	Incubation at 37°C for 1–4 h(s)	Huang and Aminoff, 1972; Koutsioulis et al., 2008
O-Glycosidase from *Streptococcus pneumoniae*	Incubation at 37°C for 1–4 h(s)	Iwase and Hotta, 1993; Yang et al., 2017

**Enzymatic/Chemical release**		

Pronase followed by solid-phase permethylation	Incubation at 55°C for 48 h(s)	Goetz et al., 2009

After being released, glycans are subjected to derivatization before matrix application and MSI analysis (Figure 1, method-I and II). Derivatization of released glycans is necessary to stabilize glycan structures and improve ionization efficiency (Holst et al., 2016). Among different approaches, the permethylation and amidation approaches are widely used in both released N-linked and O-linked glycans (Morelle et al., 2009).

The choice of a matrix is crucial in MSI analysis for tissue glycome. Homogeneous coating of the matrix allows the formation of co-crystals between matrix molecules and analytes. In addition, the matrix molecules must have the absorbing property for laser energy to aid in the soft ionization of analytes. Various matrices have been developed for glycome MSI and MS analysis listed in Table 2. The most common matrices for the MSI analysis of glycome are 2,5-dihydroxybenzoic acid (DHB) and α-cyano-4-hydroxycinnamic acid (CHCA). Usually, the matrices are sprayed on the tissue using an automated robotic sprayer such as HTX M5 Sprayer^TM^ (Stanback et al., 2021), HTX TM-Sprayer^TM^ (Shi et al., 2019), ImagePrep sprayer (Powers et al., 2014). After matrix coating, the tissue sections are sent to an MSI instruments for data acquisition and analysis. Assignments or annotation of glycans can be performed by online databases such as GlycoWorkbench and GlycanBuilder (Ceroni et al., 2007, 2008) are the tools used for the drawing of glycan structures and automatically matching these models and their theoretical fragments with the experimental mass spectra. Consortium for functional glycomics^1^ for permethylated glycans, PeakFinder tool^2^, GlycanMass^3^, GlycomeDB^4^, Glycosciences^5^, PRIDE Archive search for glycoproteins^6^, GlycoMod tool^7^ is used for N-glycan-, O-glycan-, and fragments of glycosaminoglycan annotations, METLIN^8^, and Human Metabolome Database^9^ (Kunzke et al., 2017; Yang et al., 2017; Saldova and Wilkinson, 2020).

## MSI Analysis for Glycome in the Brain

The MSI study for glycome has recently become a rapidly growing field of research. The tissue-specific or disease-associated glycome, which may act as molecular signatures for diagnosis, can be detected by direct imaging analyses and profiling of the glycome in the tissue sections. Diverse glycans are expressed in different brain tissues reported previously (Chen et al., 1998; Kleene and Schachner, 2004; Fang et al., 2016).

**TABLE 2:** List of matrices used in glycome MSI and MS analysis.

List of matrices used in glycome MSI			

Name	Synonym(s)	Application	References
2,5-dihydroxybenzoic acid	2,5-DHB	Used in MALDI-MSI analysis for N-glycans in positive ion mode	Eshghi et al., 2014; Malaker et al., 2020
α-Cyano-4-hydroxycinnamic acid and trifluoroacetic acid	CHCA	Used in MALDI-MSI analysis for N-glycans in positive ion mode	Shi et al., 2019; Stanback et al., 2021
2,6-dihydroxyacetophenone/ammonium sulfate/heptafluorobutyric acid	DHA/ammonium sulfate/HFBA	Used in MALDI-TOF-MSI analysis for sialic acids and the ceramide-associated core gangliosides in negative ion mode	Colsch and Woods, 2010
9-aminoacridine	9-AA	Used in MALDI-MSI analysis for gangliosides and native glycan fragments without prior digestion or chemical reactions in negative ion mode	Hirano-Sakamaki et al., 2015; Kunzke et al., 2017
2,5-dihydroxyacetophenone	2,5-DHAP	Used in MALDI-MSI analysis for N-glycans in positive ion mode	Heijs et al., 2020
Norharmane	NOR	Used in MALDI-MSI analysis for N-glycans in negative ion mode	Heijs et al., 2020
3-aminoquinoline	3-AQ	Used in MALDI-MSI analysis for gangliosides in negative ion mode	Zhang et al., 2016
2,6-dihydroxyacetophenone	DHA	Used in MALDI-MSI analysis for gangliosides in negative ion mode	Jackson et al., 2018
1,5-diaminonaphthalene	DAN	Used as a matrix in MALDI-MSI analysis for gangliosides in negative ion mode	Jackson et al., 2018
5-chloro-2-mercaptobenzothiazole	CMBT	Used as a matrix in MALDI-MSI analysis for gangliosides in negative ion using linear mode	Whitehead et al., 2011
3-aminoquinoline/α-cyano-4-hydroxycinnamic acid	3-AQ/CHCA	Used as a matrix in MALDI-MSI analysis for glycolipids in both negative and positive ion mode	Shimma et al., 2012
α-cyano-4-hydroxycinnamic acid and 1-methylimidazole	Ionic liquid matrix, ImCHCA	Used in MALDI-MSI analysis for gangliosides in negative ion mode	Chan et al., 2009

**List of matrices used in glycome MS**			

7-Amino-4-methylcoumarin, for fluorescence	Coumarin 120	Used in MALDI-MS analysis for monosulfated disaccharides, sulfated neutral, sialylated tri- and tetrasaccharides in negative ion mode	Dai et al., 1997
3-aminoquinoline/p-coumaric acid	3-AQ/CA	Used in MALDI-MS analysis for neutral and acidic glycans in both positive and negative ion modes	Fukuyama et al., 2014
3-Aminoquinoline/α-cyano-4-hydroxycinnamic acid	3-AQ/CHCA	Used in MALDI-MS analysis for N-glycans in negative ion mode	(Kaneshiro et al., 2011)
A mixture of 2,5-DHB and 2-hydroxy-5-methoxybenzoic acid	Super DHB	Used in MALDI-MS analysis for N-glycans in positive ion mode	Mechref and Novotny, 2010
2,5-Dihydroxybenzoic acid/N, N-dimethylaniline	DHB/DMA	Used in MALDI-MS analysis for Oligosaccharides in positive ion mode	Snovida et al., 2008
2,5-dihydroxybenzoic acid/α-Cyano-4-hydroxycinnamic acid	Binary matrices 2,5-DHB/CHCA	Used in MALDI-MS analysis for underivatized glycans and glycoproteins in positive ion mode	Laštovièková et al., 2009
2,5-dihydroxybenzoic acid/Sinapinic acid	Binary matrices 2,5-DHB/SA	Used in MALDI-MS analysis for underivatized glycans and glycoproteins in positive ion mode	Laštovièková et al., 2009
5-Chloro-2-mercaptobenzothiazole	CMBT	More sensitive than DHB for MALDI-MS analysis for high mannose N-linked glycans and peptidoglycan muropeptides in negative ion mode	Harvey, 2011
2-Acetylresorcinol; 2-Acetyl-1,3-dihydroxybenzene	2,6-dihydroxyacetophenone	Used with diammonium hydrogen citrate for MALDI-MS of PMP-labeled acidic and neutral glycans in positive ion mode	Pitt and Gorman, 1997
1,2,3-Propanetriol, matrix substance for MALDI-MS	Glycerol, Glycerin	Forms a liquid composite matrix with 4-HCCA and 3-aminoquinoline for analysis of neutral and acidic glycans in positive ion mode	Suzuki et al., 1996; Dreisewerd et al., 2006
1-methyl-beta-carboline	Harmane	Used in MS analysis of cyclodextrins and sulfated oligosaccharides combined with DHB as co-matrix in positive and negative ion modes	Nonami et al., 1998
1-Hydroxyisoquinoline; Isocarbostyril; 1-HIQ, matrix substance for MALDI-MS	1-Isoquinolinol	Used as co-matrix with DHB for MS analysis of oligosaccharides in positive ion mode	Mohr et al., 1995
Gerontine; N,N′-Bis(3-aminopropyl)-1,4-diaminobutane; Neuridine; Musculamine	Spermine	Used as co-matrix with DHB for MALDI-MS of sialylated glycans in negative ion mode	Mechref and Novotny, 1998
2-Acetylphloroglucinol	THAP	Used in MALDI analysis of acidic glycans in negative ion mode	Papac et al., 1996
6-Aza-2-thiotimine	ATT	Used in MALDI analysis of acidic glycans in negative ion mode	Lecchi et al., 1995
2-(4-Hydroxyphenylazo) benzoic acid	HABA	Used in MALDI analysis of sulfated oligosaccharides in negative ion mode	Lesur et al., 2019

While the MSI study of lipidomics in brain tissues has been conducted extensively in the last decades, glycomics was insufficient. It may be caused by challenges for laborious and delicate sample preparation using tissue sections. Diverse glycome structures, characterization, glycosylation patterns were reported by several MS methods using brain tissue in normal and disease conditions (Chen et al., 1998; Geyer et al., 2001; Liedtke et al., 2001; Ohl et al., 2003; Bleckmann et al., 2009; Breloy et al., 2012; Gizaw et al., 2016; Samal et al., 2020). In addition, the spatial information of glycome in the tissue section is lost through this glycomics procedure due to the sample homogenization (Eshghi et al., 2014).

### PNGase F-Based N-Linked Glycans MSI

**FIGURE 3:**
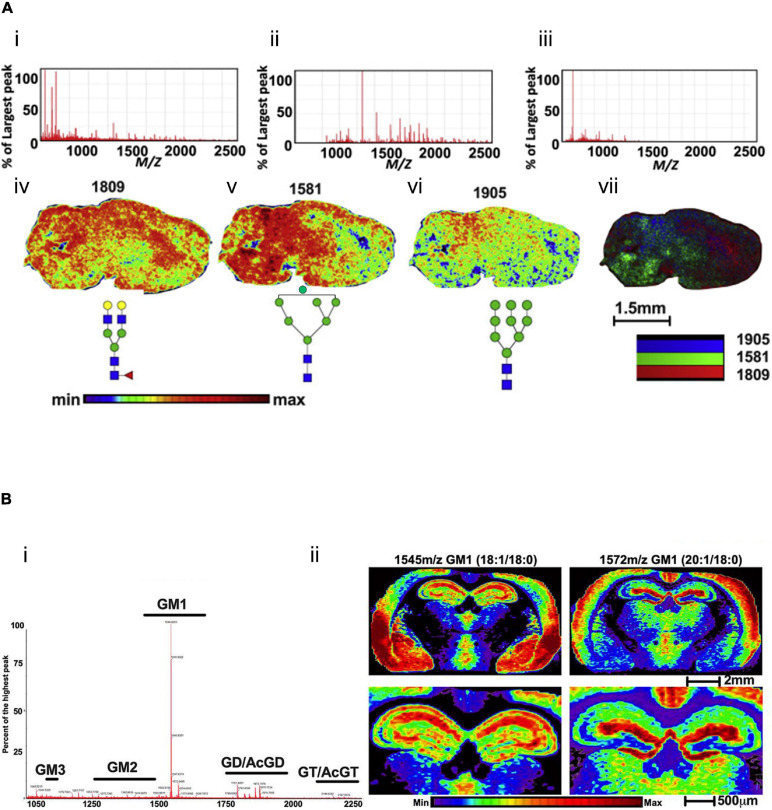
Mass spectra and ion images for glycome in mouse brain tissues. **(A)** PNGase F digestion and MALDI-MSI analysis of N-glycans in mouse brain tissue. Extracted mass spectra of released N-glycans and matrix ions for **(A-i)** both, **(A-ii)** N-glycans and **(A-iii)** matrix. Representative ion images of **(A-iv)** Hex5HexNAc4Fuc1 at *m/z* 1809, **(A-v)** Hex7HexNAc2 at *m/z* 1581, **(A-vi)** Hex9HexNAc2 at *m/z* 1905, and **(A-vii)** shows the overlay image of *m/z* 1809 (red), *m/z* 1581 (green), and *m/z* 1905 (blue). Scale bar, 1.5 mm. The mass spectra **(B-i)** and ion images **(B-ii)** of gangliosides in the mouse brain coronal section without pre-treatment of the enzyme. Gangliosides are mostly localized in the cortex and hippocampus of the mouse brain tissue section using the CHCA matrix. Scale bar, 2 mm (upper panel), 500 μm (lower panel). The figures of panels **(A,B)** are reprinted and modified from Stanback et al. (2021) and Andres et al. (2020), respectively, with free-reuse permission.

For the first time, a MALDI-MSI method has been applied to spatially profile the location and distribution of multiple N-linked glycans species in mouse brain tissues in 2013, followed by PNGase F digestion (Powers et al., 2013). This method has been developed to profile the multiple glycan species simultaneously released from intracellular organelle and cell surface glycoproteins. Thus extracted glycans were structurally analyzed by the MS method. In another study, the MSI technique was primarily used to directly analyze 42 N-glycans in FFPE mouse brain tissue sections (Eshghi et al., 2014). This procedure includes sectioning FFPE tissues, deparaffinization, rehydration, denaturing tissue proteins, releasing N-linked glycans followed by PNGase F digestion, matrix coating, and analyzing N-glycans by MALDI-MSI. The novel sub-atmospheric pressure (SubAP)/MALDI-MS system coupled with a Q Exactive HF hybrid quadrupole-orbitrap mass spectrometer was used for characterization and spatial visualization of 55 N-glycans in FFPE mouse brain tissue sections (Shi et al., 2019). PNGase F was used to promote the release of N-glycan from the brain tissues in these studies. A comprehensive MSI protocol was developed to visualize *in situ* N-glycans (at least 40 or more individual N-glycans were mapped) using FFPE and FF tissues (Drake et al., 2018). PNGase F-based N-glycan identification *in situ* multimodal MSI technique could then be applied to visualize N-linked glycans and proteins from the identical FFPE tissue section (Heijs et al., 2016). The novel combination of PNGase F with glycosidase (sialidase) was found to recognize N-glycan with greater sensitivity (Powers et al., 2013). MALDI-MSI and its advanced MALDI-2-MSI have recently been reported to show the distribution of several N-glycans (Heijs et al., 2020) in the human cerebellum from post-mortem brain tissue. In their analysis, MALDI-2-MSI has demonstrated that the sensitivity for detecting molecular [M–H]^–^ species of N-glycans increased by about three orders of magnitude, and sensitivity has increased by about a factor of 10 in positive ion mode analysis compared to the current gold standard. They also reported that enormously high structural information of complex N-glycans was obtained directly from thin tissue sections in the human cerebellum and upon low-energy collision-induced dissociation tandem MS. However, MALDI-MSI techniques are capable of visualizing hundreds of glycome with spatial information in a single brain tissue followed by PNGase F treated or non-treated sample (Figure 3A; Stanback et al., 2021), which is limited by the typical antibody staining methods. Figure 3A showed the representative ion images of Figure 3A-iv Hex5HexNAc4Fuc1 at *m/z* 1809, (Figure 3A-v) Hex7HexNAc2 at *m/z* 1581, (Figure 3A-vi) Hex9HexNAc2 at *m/z* 1905, and (Figure 3A-vii) the overlay ion image of those N-glycans in FFPE and FF mouse brain tissues by the standard MALDI-MSI protocols described by Stanback et al., 2021.

#### Limitations

PNGase F-based MSI procedures have widely been used for tissue localization of N-glycans. Special care should be taken in sample preparation steps to get efficient results. This broad specificity enzyme is applicable for a wide range of N-linked glycans MSI analysis and cannot release other glycans such as O-linked glycans.

### O-Linked Glycans MSI

Kunzke et al. (2017) published a report on O-glycans MSI analysis. They performed MSI of native and O-linked glycans fragmentation from tissue microarray and FFPE cancer tissues without prior digestion or chemical reactions using MALDI-FT-ICR-MSI (Kunzke et al., 2017). By employing their protocol, four O-linked glycans have been detected and visualized. In addition, On-tissue spatially resolved glycoproteomics strategies combined with MALDI-MSI demonstrated the global dysregulation of N-linked, O-linked glycans in canine glioma biopsies tissue (Malaker et al., 2020). This preliminary report directly links glycan imaging with intact glycopeptide identification and characterization using canine glioma FFPE tissues.

#### Limitations

N-linked glycans and their diverse heterogeneity in different biological tissues have been analyzed in detail by the different MSI techniques. However, MSI analysis for O-linked glycome has not been widely conducted in brain tissues and other organs. This is primarily attributed to the absence of a standardized enzyme with broad specificity, enabling to release of a diverse range of O-glycans, and the lack of knowledge about their O-linked counterparts. Intact O-glycans can be released chemically using a β-elimination reaction. The release of O-glycans by β-elimination or other chemical reactions is widely used for MS analysis. In this method, released glycans can be degraded, and existing knock-on effects must be overcome (Saldova and Wilkinson, 2020). The chemical method is not suitable for tissue release of glycans as it contains chemicals itself and is challenging to remove before MSI analysis (Veillon et al., 2017). O-glycans can be sequentially trimmed using a combination of exoglycosidases and an endoglycosidase (Figure 2B). Although this method can preserve a structure of proteins and activity, it may degrade the glycans (Guthrie and Magnelli, 2016, 14(2)). Thus, unlike N-linked glycans, MSI analysis of O-glycans from a thin biological tissue has not been widely investigated. Currently, no known broad specificity enzymes can release O-glycans from the protein in tissue samples. As a result, MSI analysis of O-glycans is far more complicated than for N-glycans. Universal O-glycosidases must be developed for the MSI analyses of O-glycans. Although several recombinant enzymes and chemical deglycosylation strategies are commercially available. However, more sophisticated enzymatic, chemical, and trimming methods should be developed for MSI of O-linked glycans. On the other hand, O-linked glycans can be detected and visualized as conjugated or fragmented directly from FF or FFPE brain tissues without prior digestion or chemical reactions (Kunzke et al., 2017).

### Glycolipids MSI

In addition to glycans, glycoconjugates (glycolipids) are also essential biomolecules in the brain that result from glycosylation. Apart from glycan identification, MSI techniques have been utilized to detect several glycoconjugates in tissue sections. Ganglioside-monosialic acid (GM1) is a subtype of a glycolipid known as an important area of research for studying AD. An altered GM1 to GM2/GM3 ganglioside metabolism was observed using multimodal TOF-SIMS and MALDI-based MSI strategy in the transgenic mouse model and the human brain of AD (Hirano-Sakamaki et al., 2015; Michno et al., 2019; Scholarship et al., 2020). The gangliosides (i.e., C18- or C20-sphingosine) were detected using MALDI-MSI in the frontal brain or dentate gyrus in mouse brain tissue (Sugiura et al., 2008). A prototype MSI of the iMScope (Shimadzu) has been applied to monitor the accumulation of GM2 in the hypothalamus, hippocampus, and cerebellum of the Sandhoff disease model mice (Kitakaze et al., 2016). This cutting-edge technology is also known as a mass microscope. Aside from GM1, GM2, and GM3, another glycolipid GD1 was detected and visualized spatially by MALDI-MSI in the mouse and rat brain tissue (Sugiura et al., 2008; Caughlin et al., 2017). Recently, transmission-mode geometry MALDI-MSI (t-MALDI–MSI) and MALDI-2-MSI are used to visualize several glycolipids in subcellular resolution with higher sensitivity in the cerebellum of the mouse brain tissues (Niehaus et al., 2019). N-linked, O-linked glycoconjugates, and S-linked glycopeptides have been characterized and visualized in the FFPE tissue of glioma biopsies (Malaker et al., 2020). GM1, a most abundant ganglioside family in the brain, has been visualized with higher spatial resolution in FF mouse brain tissue without enzymatic pre-treatments using SYNAPT G2-Si high definition mass spectrometry (Waters, Milford, MA, United States) (Andres et al., 2020). MALDI-FT-ICR MSI has been applied to visualize tissue localization of glycosphingolipids accumulation in Gaucher disease of mouse brain tissues (Jones et al., 2017). The unique distribution of specific glycosphingolipids family has been visualized by MALDI-MSI, DESI-MSI, and SIMS imaging techniques in different tissues, cells, and model membranes (Luberto et al., 2019). A combination of laser microdissection and liquid chromatography-electrospray ionization mass spectrometry (LC-ESI-MS) was employed for highly sensitive tissue localization of most glycolipid structural isomers in the mouse brain tissues (Ikeda and Taguchi, 2010).

Using MALDI-MSI, several types of gangliosides were spatially visualized in FF mice brain tissue without PNGase F digestion (Figure 3B; Andres et al., 2020). These analyses demonstrated that gangliosides GM1 are most abundant in the mouse brain (Figure 3B-i). In addition, a coronal image of the whole brain reveals differential cortex localization for GM1 18:1/18:0 and GM1 20:1/18:0, two of the most abundant ganglioside subtypes (Figure 3B-ii). GM1 18:1/18:0 is primarily localized in the piriform, amygdala nucleus, and striatum, where the 20:1/18:0 is localized in the anterior region of layers 1 and 2 of primary and supplemental somatosensory and the dorsal auditory areas in the brain.

#### Limitations

Currently, there is no glycans-cleaving enzyme from glycolipids. MSI of glycolipids in brain tissues as conjugated forms is the only way for imaging analysis. The dedicated MSI technologies (i.e., sensitive for conjugate molecules, MS/MS capability) are required for glycolipids detection and visualization.

Several label-free and isotopic labeling strategies have been described extensively as the most frequently used quantitative methods based on separation techniques such as LC-MS for glycome in biological samples (Zaia, 2008, 2010; Hu et al., 2013). However, MSI is a qualitative technique and considered a semi-quantitative method for biomolecules in tissues. The quantification of tissue biomolecules (lipids, proteins, and carbohydrates) by the current MSI methods is challenging due to several limitations. These include (a) the high dependence of the detected signals on the matrix deposition, (b) the MALDI ionization yield of specific target molecules, and finally, (c) the ion suppression effect on the tissue section (Hamm et al., 2012). Several attempts have been reported for quantitative analysis by MSI in tissue biomolecules. For example, semi-quantitative analysis of gangliosides in mouse brain tissues has been reported using the combination of ESI and MALDI-MSI (Zhang et al., 2016). The small tissue biomolecules such as drugs, metabolites are quantitively analyzed by the MSI method using tissue extinction calculation as a normalization factor without a labeled standard (Hamm et al., 2012). This technique is limited to small tissue biomolecules and not suited for larger biomolecules such as glycans. The quantification in tissue biomolecules by the MSI methods is under the developmental stage. Hopefully, the quantitive analysis of glycome in brain tissues by MSI methods will be achieved in the near future.

## Future Prospects of Brain MSI for Glycome

Matrix-free ionization processes such as surface-assisted laser desorption ionization (SALDI) and laser desorption ionization (LDI) are emerging as alternative MSI techniques that can provide complementary insight into molecular distributions with high ionization efficiency in the biological tissue sections. In contrast to MALDI-MSI, the matrix-free LD-MSI processes are freed from interference induced by matrix-derived ion peaks in the lower mass range. In addition, desorption ionization using through-hole alumina membrane, one of the SALDI methods, drastically lowers the sample pre-treatment time and does not require a skilled technician or dedicated instruments for matrix application, providing higher reproducibility in mass accuracy and intensity (Hasan et al., 2021).

Previously, the matrix-free LDI methods have been reported, such as gold nanoparticles (Su and Tseng, 2007; Nayak and Knapp, 2010), desorption ionization on porous silicon (Cha, 2008), nanostructured weathering steel (Etxebarria et al., 2014), silver nanostructures (Sekuła et al., 2015), chemical vapor deposited graphene surfaces (Merino et al., 2020), carbon nanoparticles and graphene nanosheets (Banazadeh et al., 2018) for glycome MS and MSI analysis. Matrix-free LDI methods have made data interpretation easier for glycome MSI analysis compare to MALDI-MSI. In addition, most LDI techniques are designed for the highly sensitive, enhanced ionization efficiency for low-mass molecules. Therefore, these LDI methods could be applicable for MSI analysis of low-mass glycolipids.

Most MSI studies are conducted in a 2D fashion where only a single section of the entire sample volume is tested. Biological processes happen within a tissue volume and can be efficiently investigated as a whole organ to achieve comprehensive information regarding spatial and molecular complexity. By registering and stacking serial tissue sections, MSI techniques can produce 3D volumes imaging demonstrating the molecular distributions in the whole tissue and organ/animal (Seeley and Caprioli, 2012). The benefit of analyzing volumetric data has led to a quick rise in the application of 3D-MSI analysis using a single sample (Vos et al., 2021). In this process, multiple 2D mass spectrometric images can be reconstructed for 3D mapping of the molecules in the entire tissue section or organ through specialized image processing software. The exploration of 3D MSI has always been challenging due to complex MSI procedures and dedicated image processing software. Some investigations regarding 3D MSI have already been done using DESI (Eberlin et al., 2010), laser ablation electrospray ionization (LAESI) (Nemes et al., 2009), MALDI (Giordano et al., 2016), and SIMS (Seeley and Caprioli, 2012). The most prominent points are the MSI analyzer and image processing software. Consider those, the TOF, Orbitrap, and FT-ICR analyzers are most suitable for 3D MSI (Makarov et al., 2006). The BioMap (Novartis, Basel, Switzerland), MATLAB (Mathworks, Natick, MA, United States), Maya software (Autodesk, Inc.), FastRBF Interpolation Toolbox (FarField Technology, Inc.), Image J are most frequently used for image data processing (Seeley and Caprioli, 2012). The potentiality of MSI techniques to create 3D representations of biomolecules in a whole organ or tissue with the image reconstruction process and high throughput processing has been reviewed (Mamun et al., 2021). The 3D volume of brain MSI for glycome has not been investigated yet. It can be an effective technique for the 3D volume of glycome imaging in entire brain tissues. The advent of 3D IMS methods has allowed for more detailed ion images of connections between signaling pathways and disease processes in various complex organs like the brain. The combination of this technology with other methods of imaging has shown the capacity to bridge the distance between non-invasive functional imaging and fundamental biology. We expect that developments of 3D MSI will continue to advance and become a significant technology in brain glycobiology in the near future.

## Conclusion

Glycan expression changes are thought to have a detrimental effect on brain function, leading to pathological brain diseases, including neurodegenerative disorders. Visualization of glycome and glycosylation patterns in complex brain tissues has been revolutionized by employing simultaneous label-free MSI techniques. The current state of MSI has generated a global snapshot of N-linked glycans in FFPE and FF brain tissue sections, showing the tissue localization, distribution, and relative abundance of glycan subtypes. MSI analysis of the counterpart, O-linked glycans, is rarely investigated due to its complicated procedures. Discovery of broad specificity O-glycosidases and development of highly sensitive MSI instruments and on-tissue quantification strategy will extend glycome research in brain tissues to analyze brain function and behavior.

## Author Contributions

MH, TK, and MS conceptualized this manuscript. All authors drafted the manuscript and have reviewed and agreed with the publication of this manuscript.

## Conflict of Interest

The authors declare that the research was conducted in the absence of any commercial or financial relationships that could be construed as a potential conflict of interest.

## Publisher’s Note

All claims expressed in this article are solely those of the authors and do not necessarily represent those of their affiliated organizations, or those of the publisher, the editors and the reviewers. Any product that may be evaluated in this article, or claim that may be made by its manufacturer, is not guaranteed or endorsed by the publisher.

## Footnotes

1
www.functionalglycomics.org

2
http://www.eurocarbdb.org/ms-tools/

3
https://web.expasy.org/glycanmass/

4
http://www.glycome-db.org

5
http://www.glycosciences.de/database/index.php

6
https://www.ebi.ac.uk/pride/

7
http://web.expasy.org/glycomod/

8
https://metlin.scripps.edu/

9
https://metlin.scripps.edu/
